# Self-perceived oral health and orofacial aesthetics of cleft patients

**DOI:** 10.1007/s40368-024-00891-w

**Published:** 2024-04-04

**Authors:** L. S. van der Knaap-Kind, L. A. Schipper, C. C. Bonifacio, E. B. Wolvius, L. Kragt

**Affiliations:** 1 Department of Oral and Maxillofacial Surgery, Special Dental Care and Orthodontics, Erasmus University Medical Center, Dr. Molewaterplein 40, 3015 GD Rotterdam, The Netherlands; 2 Department of Paediatric Dentistry, Academic Center for Dentistry Amsterdam (ACTA), Amsterdam, The Netherlands

**Keywords:** Cleft, Oral health, Orofacial aesthetics, PROMs

## Abstract

**Purpose:**

To evaluate the self-perceived oral health and aesthetics of the dentition and jaw in patients with different types of oral cleft, measured by patient-reported outcome measures (PROMs). Additionally, to compare the results of the PROMs between cleft lip and or/palate (CL/P) patients and non-affected controls.

**Methods:**

420 CL/P patients treated at the cleft team of the Erasmus Medical Center, Rotterdam, The Netherlands, were included, and 138 non-cleft patients were recruited as control-group. Patient’s perceptions were retrospectively evaluated using the CLEFT-Q Teeth for dental aesthetics at ages 8, 12 and 22, CLEFT-Q Jaw for jaw aesthetics at ages 12 and 22, and the Child Oral Health Impact Profile—Oral Symptoms Subscale (COHIP-OSS) for oral health at ages 8 and 12. One-way ANOVA was used to compare differences in oral health and aesthetic perceptions among age-groups, cleft types, as well as between cases and controls.

**Results:**

CL/P patients were significantly less satisfied than controls with their dental aesthetics (*p* = 0.001). CL/P patients reported significantly lower satisfaction on CLEFT-Q Teeth scores at ages 8 and 12, than at 22 years (*p* < 0.001). Patients with the most extensive cleft phenotype, Cleft Lip and Palate (CLAP), reported lowest satisfaction on the CLEFT-Q Teeth. No differences in perceptions of oral health nor in aesthetics of the jaw were found in the different cleft types, ages, nor in study versus control group.

**Conclusion:**

This study found differences in self-perceived dental aesthetics: CL/P patients are less satisfied than non-affected controls. CLAP patients are least satisfied, but satisfaction increases with age.

## Introduction

The condition Cleft lip and/or palate (CL/P) may affect speech, hearing, dentition and appearance of patients. Therefore, CL/P patients often require multidisciplinary care, with treatments starting from birth and continuing until young adulthood to improve patient’s appearance, function and psychosocial development (Tanaka et al. 2012). The condition and its treatment have an impact on several physiological and psychological aspects, but oral health is one of the main problems seen in CL/P patients. They have a higher risk of developing malformations in tooth shape, enamel structure and tooth alignment. Worth et al. (2017) showed that CL/P patients are more susceptible to the development of dental caries lesions than patients without CL/P. This higher caries risk is not only because oral hygiene is complicated in CL/P patients due to several dental anomalies, but also due to a  deviant anatomy.

The influence of the CL/P condition and its treatment on dental health is traditionally measured with clinician reported outcomes, such as indexes on caries experience and malocclusion (Heliövaara et al. 2017; Long et al. 2011; Mølsted et al. 2005). While these objective measurements remain important outcome measures, several life aspects are preferably measured subjectively. Moreover, correlating objective and subjective measurements seem to complement each other (van der Knaap-Kind et al. 2024). For a more comprehensive assessment of oral health, it is becoming increasingly relevant to obtain the patients’ perspective on their health and quality of life (Wong Riff et al. 2018). Though CL/P patients have reported a lower oral health related quality of life compared to non-cleft patients (Rando et al. 2018), research about the effects of the cleft treatment on patient-reported outcome measures (PROMs) is scarce.

In 2015, the International Consortium for Health Outcomes Measurement (ICHOM) developed the Standard Set for Cleft Lip and Palate (ICHOM-SCS). ICHOM-SCS is a set combining PROMs and clinician-reported measures to compare and evaluate the outcomes of different treatment protocols. Their main goal is to improve the quality of care and promote the transition to value-based health care. The dentistry related PROMs that are used in the ICHOM-SCS are the Child Oral Health Impact Profile—Oral Symptoms Subscale (COHIP-OSS), CLEFT-Q Teeth and CLEFT-Q Jaw (Bittar et al. 2018; Allori et al. 2017).

Though the effort has been made to create and implement datasets like ICHOM-SCS, the translation of the results into clinical practice has not been further explored. Therefore, the aim of this study is to evaluate the oral health and aesthetic perceptions of dentition and jaw in patients with different cleft types, at different ages. Additionally, the outcomes of CL/P patients are compared with the outcomes of a non-cleft control group to evaluate differences in outcomes.

## Material and methods

### Ethical approval

This study was approved by the Medical Ethics Review Committee of the Erasmus University Medical Center, Rotterdam, The Netherlands, number: MEC-2019-0535.

### Study population

#### Study group

In this study 420 patients with CL/P have been included. The patient group was recruited at the outpatient clinic of the Erasmus Medical Center (Erasmus MC), Rotterdam, The Netherlands, during CL/P specific consultation hours. Since 2015, the ICHOM data collection is an integral part of the treatment plan for CL/P patients in the Erasmus MC. Generally, all CL/P patients observed and treated by the Erasmus MC cleft team are eligible for participation in ICHOM data collection. The current study includes all CL/P patients that visited the cleft team at the ages on which the questionnaires concerning oral health and aesthetic perceptions of dentition and jaw were applied (8, 12, and 22 years old). ICHOM has defined the following ranges for these age groups: the 8 years group is aged between 8 years, 0 months and 9 years, 11 months; the 12 years group ranges from 12 years, 0 months to 12 years, 11 months; and finally, the 22 years group ranges from 22 years 0 months to 22 years 11 months.

Patients who were not able to complete the questionnaires due to cognitive impairments or language barriers, were excluded from ICHOM data collection. Cleft types were classified as Cleft Lip (CL), Cleft Palate (CP), Cleft Lip and Alveolus (CLA) and Cleft Lip and Palate (CLAP).

#### Control group

The control group was randomly selected from two dental practices (SPI and AER) and from the Oral and Maxillofacial Surgery department of the Erasmus MC. In total, 138 non-cleft patients aged 8, 12 or 22 years were asked to participate in the current study. The age groups of the controls corresponded with the ICHOM age groups. The patients had no syndrome or remarkable facial deformity and there was no language barrier that impaired them to complete the questionnaires.

An a priori power analysis was conducted using G*Power version 3.1.9.7 (Faul et al. 2007) for sample size estimation. With a total of 300 CLP patients and 120 controls, a two-sided alpha of 0.05, this study has a power of 0.8 to show the expected effect size of d = 0.28.

However, in both study and control group more patients could be included. With a total of 420 CL/P patients and 138 controls, and a two-sided alpha of 0.05, this study has a power of 0.92 to detect an effect size of d = 0.28.

### Data collection procedure

Patients at the age 8 and 12 were asked to fill out the questionnaire together with supervision from their caregiver, 22 year-old patients filled in the questionnaires without supervision. Ombashi et al. (2023) concluded that the large majority of questionnaires in age-groups 8 and 12 years was indeed filled in by patient together with at least one caregiver, and they found no significant differences in mean scores of the CLEFT-Q’s per reporter type.

#### Study group

Amongst other questionnaires, the CLEFT-Q Teeth, CLEFT-Q Jaw and COHIP-OSS were sent to the patients per email prior to the cleft team appointment. The majority of the patients filled in the questionnaire at home. A few patients who did not finish the questionnaire before the appointment were asked to complete the questionnaire afterwards.

All data were digitally stored on the server of the Erasmus MC. Data for this study were collected between November 2015 and December 2019.

#### Control group

The control group completed the questionnaires on paper. Patients of the control group were asked by the front desk assistant to fill out the questionnaires in the waiting area. All data were anonymously collected, they were scanned and digitally saved. Data were collected between September 2019 and February 2020.

#### Questionnaires

The outcome measures, at the specific ages and for selected cleft types, that were included in the ICHOM-SCS have been selected by expert consensus (both cleft clinicians and patients). This process of creating a standardized set of outcome measures for Cleft Lip and Palate was elaborately described by Allori et al. (2017). The CLEFT-Q is a validated questionnaire to evaluate treatment outcomes in patients with CL/P (Klassen et al. 2018; Wong Riff et al. 2017; Tsangaris et al. 2017). It is developed for international use, is CL/P-specific and applicable for patients aged between 8 and 29 years old (Klassen et al. 2012; Wong Riff et al. 2017). The CLEFT-Q includes 12 subscales relevant for CL/P patients, like hearing, speaking, appearance and school life. All PROMs were translated from the original English version into the native language of The Netherlands. The translation and cultural adaptation guidelines of the International Society for Pharmacoeconomics and Outcomes Research were followed [Tsangaris et al. 2018].

In the current study, three questionnaires regarding the patient’s perception of oral health and the aesthetics of teeth and jaw were used: the CLEFT-Q Teeth, CLEFT-Q Jaw and the Child Oral Health Impact Profile–Oral Symptoms Subscale (COHIP-OSS).

The CLEFT-Q Teeth, was used to assess the dental aesthetics, this questionnaire contains 8 questions that are answered on a five-point Likert scale (0 = ‘never’, to 4 = ‘constantly’). The questions are positively worded, thus higher scores equal higher satisfaction, with a total sum score ranging from 0 to 32. The CLEFT-Q Teeth was applied at the ages of 8, 12 and 22 years.

Another CLEFT-Q subscale, the CLEFT- Q Jaw, was used in this study to assess the aesthetics of the jaw. This questionnaire has 7 questions, answered on a five-point Likert scale (0 = ’never’, to 4 = ’constantly’). The questions are positively worded, therefore higher scores equal higher satisfaction, with a total sum score ranging from 0 to 28. The CLEFT-Q Jaw is applied at the ages of 12 and 22 years.

The Child Oral Health Impact Profile (COHIP) is a validated tool to assess the impact of different oral conditions (including CL/P) on the oral health-related quality of life. The COHIP has been designed to be administered to children ranging from 8 to 15 years old (Broder et al. 2007; Wilson-Genderson et al. 2007). The COHIP has four subscales: Oral Health, Functional Well-Being, Social-Emotional Well-Being and School Environment. The COHIP-OSS, which is a shortened version of the Oral health subscale, focusses on the perceived oral health (Broder and Wilson-Genderson 2007). The COHIP-OSS contains 5 questions that are answered on a five-point Likert scale (0 = ‘never’, to 4 = ‘constantly’). Lower scores equal higher satisfaction, with a total sum score ranging from 0 to 20. The COHIP-OSS was applied at the ages of 8 and 12 years, but was not used for patients born with CL, see Table 1.

**Table 1:** ICHOM moments of measurement

Measure	Moment of measurement	Cleft type
CLEFT-Q Teeth	Age 8, 12, 22	CL, CP, CLA, CLAP
CLEFT-Q Jaw	Age 12, 22	CL, CP, CLA, CLAP
COHIP-OSS	Age 8, 12	CP, CLA, CLAP

### Statistical analysis

Descriptive statistics were used to describe the study population. Chi square tests were used to evaluate differences between study and control group. The sum scores of the CLEFT-Q Teeth, CLEFT-Q Jaw and COHIP-OSS were each individually calculated and presented as mean values with standard deviations (sd). One-way ANOVAs including post-hoc analyses were used to measure differences in sum scores between age-groups, gender, cleft types and between CL/P patients and controls. All data were processed and analyzed using IBM SPSS Statistics for Windows, Version 25.0. Armonk, NY, USA.

## Results

This study was conducted with 558 participants. There was a total of 420 participants in the CL/P group (75.3%), and 138 participants in the control group (24.7%). Table 2 shows the characteristics of the participants. The most common cleft type in all age groups was the CLAP phenotype (49.3%), followed by the CP phenotype (32.4%). The participants were divided into the 3 age categories as follows: 230 8-year-olds (41.2%), 195 12-year-olds (35.0%), and 133 22-year-olds (23.8%). In this study, 55.6% of the participants (n = 310) were male and 44.4% female (n = 248). There was no difference in age distribution between study and control group (χ^2^ (2,558) = 3.934, *p* = 0.140), but there was a difference in gender between study and control group (χ^2^ (1,558) = 6.256, *p* = 0.012). A male majority in the current CL/P group corresponds with literature on CL/P prevalence (Pool et al. 2021).

**Table 2:** Characteristics of the population

	CL (n = 38)	CP (n = 136)	CLA (n = 39)	CLAP (n = 207) %	Control (n = 138) %	p-value*
Age group						0.140
8	17 (45)	54 (40)	14 (36)	87 (42)	58 (42)	
12	11 (29)	51 (37)	16 (41)	77 (37)	40 (29)	
22	10 (26)	31 (23)	9 (23)	43 (21)	40 (29)	
Gender						0.014*
Male	21 (55)	59 (43)	24 (62)	142 (69)	64 (46)	
Female	17 (45)	77 (57)	15 (38)	65 (31)	74 (54)	

*CL*  Cleft lip, *CP*  Cleft Palate, *CLA* Cleft lip and alveolus, *CLAP*  Cleft lip alveolus and palate

*Significant difference. p < 0.05 was stated as significant, based on chi-square test

### CLEFT-Q Teeth

The CLEFT-Q Teeth was filled in by 411 CL/P patients, they had an average sum score of 20.96 ± 5.73. There were 135 controls with an average sum score of 22.96 ± 5.96. Outcomes per cleft type and per age group are presented in Table 3.

**Table 3:** Mean (SD) and number of outcomes for CLEFT-Q Teeth

	CL	CP	CLA	CLAP	p-value	Control	p*-*value cleft vs control
Age group							
8	20.18 (6.54), n = 17	20.12 (6.59), n = 52	21.69 (5.02), n = 13	18.29 (4.75), n = 85	0.038 *	22.73 (5.88), N = 56	< 0.001 *
12	22.64 (4.88), n = 11	21.08 (5.85), n = 50	22.53 (5.34), n = 15	19.87 (5.22), n = 75	0.399	23.49 (5.75), n = 39	0.007 *
22	26.10 (6.82), n = 10	24.61 (5.65), n = 31	24.56 (4.16), n = 9	23.42 (5.40), n = 43	0.146	22.75 (6.38), n = 40	0.232
Total	22.45 (6.50), n = 38	21.53 (5.96), N = 133	22.73 (4.96), n = 37	19.96 (5.40), N = 203	0.003 *	22.96 (5.96), n = 135	< 0.001 *

Outcomes are given per age-group. One-way ANOVAs were performed. *CL*  Cleft Lip, *CP*  Cleft Palate, *CLA*  cleft lip and alveolus, *CLAP* Cleft lip alveolus and palate

^*^Significant difference. p < 0.05 was stated as significant

There was a statistically significant difference in the sum score of the CLEFT-Q Teeth among cleft type groups (F (3407) = 4.638, *p* = 0.003). Post-hoc analysis showed that patients with the most extensive cleft phenotype (CLAP) reported significantly lower CLEFT-Q Teeth scores compared to CLA cleft type (*p* = 0.039). However, no significant differences were found between CLAP and CL (*p* = 0.80) nor between CLAP and CP (*p* = 0.81).

There was a statistically significant difference in CLEFT-Q Teeth sum scores between age groups in the CL/P group (F (2408) = 24.485, *p* < 0.001) but not in the control group (F (2132) = 0.216, *p* = 0.806). CL/P patients reported overall significantly lower CLEFT-Q Teeth scores at ages of 8 and 12 years, than at 22 years (both *p* < 0.001). No significant differences in mean CLEFT-Q Teeth sum score between males and females were found in the CL/P group (F (1409) = 0.927, *p* = 0.336), and this was similar in the control group (F (1133) = 2.800, *p* = 0.097).

There was a statistically significant difference in CLEFT-Q Teeth sum score between CL/P group and control group (F (1544) = 12.240, *p* < 0.001). Analysis showed that the control group scored significantly higher than the CL/P group in the CLEFT-Q Teeth scale at the ages of 8 (*p* < 0.001) and 12 years (*p* = 0.006). At the age of 22 the difference in CLEFT-Q Teeth scores between the CL/P group and the control group disappeared (*p* = 0.232).

### CLEFT-Q Jaw

The CLEFT-Q Jaw was filled in by 241 CL/P patients, they had a mean sum score of 22.51 ± 4.50. The control group had a mean sum score of 21.88 ± 6.02. Outcomes per cleft type and per age group are presented in Table 4.

**Table 4:** Mean(SD) and number of outcomes for CLEFT-Q Jaw

	CL	CP	CLA	CLAP	p-value	Control	p-value cleft vs control
Age group							
12	22.64 (3.906), n = 11	23.16 (5.080), n = 49	23.40 (3.460), n = 15	21.86 (4.753), n = 73	0.415	23.45 (2.834), n = 40	0.263
22	24.80 (3.490), n = 10	22.10 (4.369), n = 31	23.44 (3.432), n = 9	22.09 (4.270), n = 43	0.246	20.30 (8.176), n = 40	0.023 *
total	23.67 (3.786), n = 21	22.75 (4.817), n = 80	23.42 (3.374), n = 24	21.95 (4.562), n = 116	0.223	21.88 (6.022), n = 80	0.318

Outcomes are given per age-group. One-way ANOVAs were performed. CL cleft lip *CP*  cleft palate, *CLA *  cleft lip and alveolus, *CLAP* = Cleft lip alveolus and palate

^*^Significant difference. p < 0.05 was stated as significant

There was no statistically significant difference in the sum score of the CLEFT-Q Jaw among cleft types (F (3237) = 1.473, *p* = 0.223) nor between males and females (F(1239) = 0.950, *p* = 0.331), nor between age groups (F (1,39) = 0.000, *p* = 0.987). There was no statistically significant difference in sum score of the CLEFT-Q Jaw between man and woman in the control group (F (1,78) = 0.061, *p* = 0.806), but there was a statistically significant difference between age groups in the control group (F (1,78) = 5.805, *p* = 0.018).

Moreover, there was no statistically significant difference between CL/P group and control group in general (F (1319) = 1.001, *p* = 0.318), but with a focus on 22 year-olds group only, the CL/P group reported a significantly higher CLEFT-Q Jaw sum score than the control group (*p* = 0.023).

### COHIP-OSS

The COHIP-OSS was filled in by 294 CL/P patients with an average sum score of 4.96 ± 3.27, which was not significantly different with the average sum score of the control group (n = 97, mean sum score of 5.27 ± 3.346) (F (1389) = 0.642, *p* = 0.423). Outcomes per cleft type and per age group are presented in Table 5.

**Table 5:** Mean (SD) and number of outcomes for COHIP-OSS

	CP	CLA	CLAP	p-value	Control	p-value cleft vs control
Age group						
8	5.09 (3.80), n = 54	4.57 (2.95), n = 14	5.67 (3.23), n = 85	0.413	5.32 (3.30), n = 57	0.924
12	4.72 (3.02), n = 50	3.87 (3.18), n = 16	4.52 (3.09), n= 75	0.634	5.20 (3.46), n = 40	0.229
Total	4.91 (3.43), n = 104	4.20 (3.04), n = 30	5.13 (3.21), n = 160	0.355	5.27 (3.35), n = 97	0.423

Outcomes are given per age-group. One-way ANOVAs including post-hoc analyses were performed

^*^Significant difference. p < 0.05 was stated as significant

There was no statistically significant difference in sum score of the COHIP-OSS in the study group among cleft type groups (F (2,291) = 1.038, *p* = 0.355), nor between gender (F (1292) = 3.246, *p* = 0.073). However, there was a statistically significant difference in sum score of the COHIP-OSS between age groups within the CL/P group (F (1,292) = 4.995, *p* = 0.026).

There was no statistically significant difference in sum score of the COHIP-OSS between age groups in the control group (F (1,95) = 0.028, *p* = 0.868), nor between boys and girls of the control group (F (1,95) = 0.356, *p* = 0.552).

## Discussion

This study showed that a cleft anomaly has an impact on the perception of dental aesthetics as our results indicate a correlation between the extent of the cleft with unfavourable dental aesthetic scores. It is advised to discuss this possible unsatisfactory situation with patients and investigate if a (restorative) solution is desirable and possible. Moreover, our results suggest that the perception of dental aesthetics among CL/P patients improves with age. However, cleft type as well as gender and age did not contribute significantly to the perception of oral health and perceived aesthetics of the jaw neither in the CL/P group nor was there a difference with the control group. In general, to address treatment needs individually, healthcare providers should consider the patient’s age, cleft type and patient’s perceptions next to clinical outcomes.

Differences between CL/P patients and non-affected patients could have been expected on all 3 aspects: aesthetics of the teeth, aesthetics of the jaw and oral health. After all, CL/P patients experience more often dental anomalies, more frequently a deviating dental occlusion and more caries than non-affected persons (Williams et al. 2001). But in this study, the CL/P patients perceive only their dental aesthetics as lower compared to their non affected peers.

Patients with a CLAP phenotype were the least satisfied with their dental aesthetics, compared to the other cleft types. This was also seen in the study of Wong Riff et al. (2019), where CLAP resulted in the lowest score in the CLEFT-Q Teeth, compared with other cleft phenotypes. Comparably, in the study of Klassen et al. (2018), a lower CLEFT-Q teeth score was reported by CL/P patients with palate involvement. Concerning the CLEFT-Q Jaw; in the study of Wong Riff (2019), the bilateral CLAP and bilateral CL phenotypes have the lowest satisfaction. Also, in the present study the CLEFT-Q Jaw sum scores were lowest in the CLAP group, but this difference was not significant. Corresponding to the perception of the patient, is CLAP the most severe CL/P phenotype from a clinical point of view. It might be more important to discuss aesthetic impairment as well as aesthetic treatment effects in the CLAP phenotype than in the other CL/P phenotypes.

In most cases, the 22 years measurement is the moment that all CL/P treatments are finalized, meaning most of the orthodontic treatments, surgeries and other cleft treatments are completed at the age of 22. CL/P patients at the age group of 22 years report to be more satisfied with their dental aesthetics than the CL/P patients at the younger age groups. This difference could be explained by the cleft treatment that the patient received, as one of the treatment goals is to improve dental appearance. According to the CLEFT-Q Jaw, the 22 year-olds CL/P were even more satisfied than the 22 year-olds control group. This is an unexpected result as malocclusions are more prevalent among CL/P patients, yet an explanation might be that CL/P patients are very happy after finalizing the cleft treatment trajectory, that also included an option for aesthetic jaw surgery. Furthermore, this might suggest that the CLEFT-Q Jaw is an important PROM for CL/P patients, as own experiences are relative. The difference in de CLEFT-Q Jaw scores at age 12 and 22 is not significant, this might suggest that the CLEFT-Q Jaw is irrelevant at a younger age.

Both CL/P patients as non-affected controls reported less oral health problems via the COHIP-OSS at the age of 8, than at the age of 12. This could be explained by the corresponding dental situation, 12 years olds are usually in the second transition phase or even already wearing orthodontic braces. With a very high standard deviation and low sum scores, the COHIP-OSS hardly seems to detect any problems. These findings might suggest that the COHIP-OSS is not so relevant for specifically CL/P patients, which was also concluded before by van der Knaap-Kind et al. in 2024. One limitation of the current study is that COHIP-OSS sum scores are used, but this can be debated as RASCH analysis showed no clinical hierarchy in the 5 items of the COHIP-OSS. Therefore, it should be preferably used as a problem checklist rather than a scale, meaning each item should be interpreted individually (Apon et al. 2021).

For future research it is advised to include information on use of orthodontic appliances, caries experience and malocclusion of both study and control groups to enable a better comparison between these groups. Also, in the current study there was a difference in location and method of filling out the questionnaire: at home versus the waiting area, and electronic versus in paper. To increase reliability for future research it would be better to use similar location and method for both groups. Finally, in a few years when results are available from a longer period, this study could present repeated measures per patient and also more patients per cleft type can be presented.

## Conclusion

Considering any limitations of the present questionnaire study in Dutch CL/P children, adolescents and young adults it has been shown that the cleft severity influences the perception of dental aesthetics, especially at younger age. Particularly if patient-reported outcome measures are not used during the cleft treatment trajectory, clinicians should be aware that CL/P patients perceive their dental aesthetics different than non-affected persons. This study carefully suggests that assessment of perceived dental aesthetics is relevant for CL/P patients, the assessment of perceived jaw aesthetics is more relevant at an older age and perceived oral health might be less relevant for the CL/P treatment trajectory.

## Data Availability

Data are not publicly available, but can be accessed upon request.

## References

[CR1] Allori AC, Kelley T, Meara JG, Albert A, Bonanthaya K, Chapman K (2017). A standard set of outcome measures for the comprehensive appraisal of cleft care. Cleft Palate Craniofac J.

[CR2] Apon I, van Leeuwen N, Allori AC, Rogers-Vizena CR, Koudstaal MJ, Wolvius EB, Cano SJ, Klassen AF, Versnel SL (2021). Rasch analysis of patient- and parent-reported outcome measures in the international consortium for health outcomes measurement standard set for cleft lip and palate. Value Health.

[CR3] Bittar PG, Carlson AR, Mabie-DeRuyter A, Marcus JR, Allori AC (2018). Implementation of a standardized data-collection system for comprehensive appraisal of cleft care. Cleft Palate Craniofac J.

[CR4] Broder HL, Wilson-Genderson M (2007). Reliability and convergent and discriminant validity of the child oral health impact profile (COHIP Child's version). Community Dent Oral Epidemiol.

[CR5] Broder HL, McGrath C, Cisneros GJ (2007). Questionnaire development: face validity and item impact testing of the child oral health impact profile. Community Dent Oral Epidemiol.

[CR8] Faul F, Erdfelder E, Lang AG, Buchner A (2007). G*Power 3: a flexible statistical power analysis program for the social, behavioral, and biomedical sciences. Behav Res Methods.

[CR9] Heliövaara A, Küseler A, Skaare P, Shaw W, Mølsted K, Karsten A, Brinck E, Rizell S, Marcusson A, Sæle P, Hurmerinta K, Rønning E, Najar Chalien M, Bellardie H, Mooney J, Eyres P, Semb G (2017). Scandcleft randomised trials of primary surgery for unilateral cleft lip and palate: 6 dental arch relationships in 5 year-olds. J Plast Surg Hand Surg..

[CR10] Klassen AF, Tsangaris E, Forrest CR, Wong KW, Pusic AL, Cano SJ, Syed I, Dua M, Kainth S, Johnson J, Goodacre T (2012). Quality of life of children treated for cleft lip and/or palate: a systematic review. J Plast Reconstr Aesthet Surg.

[CR11] Klassen AF, Riff KWW, Longmire NM, Albert A, Allen GC, Aydin MA, Baker SB, Cano SJ, Chan AJ, Courtemanche DJ, Dreise MM, Goldstein JA, Goodacre TEE, Harman KE, Munill M, Mahony AO, Aguilera MP, Peterson P, Pusic AL, Slator R, Stiernman M, Tsangaris E, Tholpady SS, Vargas F, Forrest CR (2018). Psychometric findings and normative values for the CLEFT-Q based on 2434 children and young adult patients with cleft lip and/or palate from 12 countries. CMAJ.

[CR12] Long RE, Hathaway R, Daskalogiannakis J, Mercado A, Russell K, Cohen M, Semb G, Shaw W (2011). The Americleft study: an inter-center study of treatment outcomes for patients with unilateral cleft lip and palate part 1 principles and study design. Cleft Palate Craniofac J.

[CR13] Mølsted K, Brattström V, Prahl-Andersen B, Shaw WC, Semb G (2005). The Eurocleft study: intercenter study of treatment outcome in patients with complete cleft lip and palate Part 3: dental arch relationships. Cleft Palate Craniofac J..

[CR14] Ombashi S, van Roey VL, Okkerse JME, van Veen-van M, der Hoek EEB, van Oers-Hazelzet AB, van der Molen M, Versnel SL (2023). Who should fill out a pediatric PROM? psychometric assessment from a clinical perspective in 567 children with a cleft. Face.

[CR15] Pool SMW, der Lek LMV, de Jong K, Vermeij-Keers C, Mouës-Vink CM (2021). Embryologically based classification specifies gender differences in the prevalence of orofacial cleft subphenotypes. Cleft Palate Craniofac J.

[CR16] Rando GM, Jorge PK, Vitor LLR, Carrara CFC, Soares S, Silva TC, Rios D, Machado MAAM, Gavião MB, Oliveira TM (2018). Oral health-related quality of life of children with oral clefts and their families. J Appl Oral Sci.

[CR18] Tanaka SA, Mahabir RC, Jupiter DC, Menezes JM (2012). Updating the epidemiology of cleft lip with or without cleft palate. Plast Reconstr Surg.

[CR19] Tsangaris E, Wong Riff KWY, Goodacre T, Forrest CR, Dreise M, Sykes J, de Chalain T, Harman K, O'Mahony A, Pusic AL, Thabane L, Thoma A, Klassen AF (2017). Establishing content validity of the CLEFT-Q: a new patient-reported outcome instrument for cleft lip/palate. Plast Reconstr Surg Glob Open.

[CR20] Tsangaris E, Wong Riff KWY, Dreise M (2018). Translation and cultural adaptation of the CLEFT-Q into Arabic, Dutch, Hindi, Swedish, and Turkish. Eur J Plast Surg.

[CR22] van der Knaap-Kind LS, Ombashi S, Van Roey V, Kragt L, Peterson P, Jabbari F, Wolvius EB, Versnel SL (2024). Evaluation and recommendations of the oral health, oral function, and orofacial aesthetics-related measures of the ICHOM Standard Set for Cleft Lip and Palate. Int J Oral Maxillofac Surg.

[CR23] Williams AC, Bearn D, Mildinhall S, Murphy T, Sell D, Shaw WC, Murray JJ, Sandy JR (2001). Cleft lip and palate care in the United Kingdom–the clinical standards advisory group (CSAG) study part 2: dentofacial outcomes and patient satisfaction. Cleft Palate Craniofac J.

[CR24] Wilson-Genderson M, Broder HL, Phillips C (2007). Concordance between caregiver and child reports of children's oral health-related quality of life. Community Dent Oral Epidemiol.

[CR25] Wong Riff KW, Tsangaris E, Goodacre T, Forrest CR, Pusic AL, Cano SJ, Klassen AF (2017). International multiphase mixed methods study protocol to develop a cross-cultural patient-reported outcome instrument for children and young adults with cleft lip and/or palate (CLEFT-Q). BMJ Open.

[CR26] Wong Riff KWY, Tsangaris E, Goodacre TEE, Forrest CR, Lawson J, Pusic AL, Klassen AF (2018). what matters to patients with cleft lip and/or palate: an international qualitative study informing the development of the CLEFT-Q. Cleft Palate Craniofac J.

[CR27] Wong Riff KWY, Tsangaris E, Forrest CR, Goodacre T, Longmire NM, Allen G, Courtemanche DJ, Goldstein J, O'Mahony A, Pusic AL, Slator R, Swan MC, Thoma A, Vargas F, Klassen AF (2019). CLEFT-Q: detecting differences in outcomes among 2434 patients with varying cleft types. Plast Reconstr Surg.

[CR28] Worth V, Perry R, Ireland T, Wills AK, Sandy J, Ness A (2017). Are people with an orofacial cleft at a higher risk of dental caries? A systematic review and meta-analysis. Br Dent J.

